# Phase I multi-center clinical and biomarker study of the dual-action androgen receptor inhibitor ONCT-534

**DOI:** 10.1007/s10637-026-01600-8

**Published:** 2026-03-03

**Authors:** Matthew R. Chrostek, James Robinson, Rajesh Krishnan, Evan Y. Yu, Luke T. Nordquist, Andrae L. Vandross, Mohamad A. Salkeni, Jennifer Schehr, Matthew Mannino, Alyssa Hintz, Jacob Caceres, Manushi Vatani, Hamid Emamekhoo, Peter Nelson, Chris Markey, Elisabeth Coates, James Breitmeyer, Lawrence Fong, Johann S. De Bono, Joshua M. Lang, Shuang G. Zhao

**Affiliations:** 1 Department of Human Oncology, University of Wisconsin-Madison, Madison, WI USA; 2 Oncternal Therapeutics, Inc., San Diego, CA USA; 3 Fred Hutchinson Cancer Center, Seattle, WA USA; 4 Urology Cancer Center, PC, Omaha, NE USA; 5 NEXT Oncology, Austin, TX USA; 6 NEXT Oncology, Fairfax, VA USA; 7 Carbone Cancer Center, University of Wisconsin-Madison, Madison, WI USA; 8 Department of Medicine, University of Wisconsin-Madison, Madison, WI USA; 9 The Royal Marsden Hospital NHS Foundation Trust, London, UK

**Keywords:** Dual-action androgen receptor inhibitor, MCRPC, Hormonal therapies

## Abstract

ONCT-534 is a dual-action androgen receptor inhibitor (DAARI) that combines AR antagonism and degradation via N-terminal domain binding, additionally targeting AR splice variants. In this study patients received ONCT-534 daily ranging from 40 to 1200 mg. The primary objectives were safety and dose-limiting toxicities (DLTs). Additional objectives included antitumor activity and AR signaling biomarkers. Adverse events (AEs) were assessed within the first 28 days for DLTs. Clinical activity was evaluated by PSA and radiographic progression per PCWG3 and RECIST v1.1. The trial was stopped early and not all patients were evaluated per protocol. Twenty-one patients received ONCT-534 across six doses. The most common AEs were anemia, back pain, and fatigue. While no DLTs were observed within 28 days, Grade 3/4 AEs occurred later in 9 patients (anemia most frequently). One patient discontinued due to treatment-related AE. No radiographic or PSA-based responses were observed; however, three patients showed PSA declines at week 4, with one achieving a PSA50 at time of study closure. Of 10 patients with baseline AR protein data, the median change was − 40.59% in AR protein levels at week 4. In the 300 mg cohort, 3 patients showed concordant decreases in AR protein and AR gene expression. Here, PSA levels correlated positively with AR target gene and AR gene expression. ONCT-534 had acceptable safety and demonstrated biological activity through AR protein degradation and AR signaling suppression in mCRPC patients. Although clinical responses weren’t observed, these findings provide proof-of-concept for examining AR protein changes in patients receiving DAARIs.

**Supplementary Information:**

The online version contains supplementary material available at 10.1007/s10637-026-01600-8.

## Introduction

Hormonal therapies targeting androgen signaling forms the backbone of systemic therapies for prostate cancer. Androgen deprivation therapy (ADT) is used both in early-stage and advanced prostate cancer (e.g. leuprolide, relugolix, bicalutamide). Advanced disease is typically treated by adding AR signaling inhibitors (ARSIs, e.g. enzalutamide, apalutamide, darolutamide, abiraterone). While castration-sensitive prostate cancers (CSPC) are initially responsive to ADT, nearly all patients develop castration-resistant prostate cancer (mCRPC) eventually [1]. Even in CRPC, rising PSA levels suggest AR remains a principal driver of disease [2]. Common molecular drivers of treatment resistance include androgen receptor (AR) genomic and epigenomic alterations [3–5], constitutively active AR splice variants [6, 7], and lineage-state transitions [1, 8–10].

New therapeutic strategies to overcome these resistance mechanisms by directly degrading AR have emerged. AR proteolysis targeting chimeras are heterobifunctional molecules with 2 recruiting ligands connected by a linker. One ligand binds to a site on AR and the other recruits E3 ligase, forming a ternary complex upon binding of both ligands. E3 ligase then facilitates poly-ubiquitination, which leads to the breakdown of AR [11]. By directly degrading AR, this class of drug can act independent of AR function or AR expression levels [12] and may thus overcome a number of AR-dependent mechanisms of drug resistance seen in CRPC patients that are not addressable by ARSIs. However, this class of compounds typically relies exclusively on significant degradation of the AR and may not trigger an effect as rapidly as ARSIs.

ONCT-534 (previously known as GTx-534 or UT-34) is a dual-action androgen receptor inhibitor (DAARI) that acts across cell types in the body, with a novel mechanism of AR inhibition via both receptor antagonism and degradation. It binds to the AR-activation function-1 domain (AF-1) of the N-terminal, directly antagonizing AR function, and also engaging the E3 ubiquitin ligase, leading to intracellular degradation of bound AR protein. ONCT-534 has demonstrated preclinical activity in prostate cancer models against both wild-type AR and altered AR, including AR amplification, mutations in the AR Ligand Binding Domain, and splice variants with loss of the AR LBD [13]. In xenograft models, ONCT-534 also demonstrated inhibitory action in vivo [14]. Here, we present the first-in-human results of ONCT-534, from a phase I clinical trial studying the safety and preliminary activity of ONCT-534 in mCRPC patients.

## Methods

### Study design and patient selection

This phase I, multi-center (USA- and UK-based sites), open-label, dose escalation study was designed to evaluate the safety, tolerability, and dose limiting toxicity of ONCT-534 in patients with relapsed or refractory histologically confirmed metastatic castration-resistant prostate cancer (mCRPC) that is also resistant or refractory to at least one ARSI, with the purpose of informing two dose levels or schedules for a phase II study (ClinicalTrials.gov: NCT05917470 registered 2023–06–23). Secondary and exploratory objectives included assessment of antitumor activity and assessment of genes and protein involved in androgen receptor signaling respectively. The study protocol was approved by institutional review boards before patient recruitment and conducted in accordance with the Declaration of Helsinki, International Council for Harmonization Good Clinical Practices Guidelines, and local regulations governing the conduct of clinical studies. Each patient provided informed consent before enrollment.

Study eligibility criteria are described in the Data Supplement. Using an adaptive Bayesian Optimal Interval (BOIN) design, subjects were initially assigned to one of five ONCT-534 dose cohorts, 40, 80, 160, 300, or 600 mg, administered orally once per day. The number of dose cohorts was amended to 6 with the addition of a 1200 mg cohort at the discretion of the Safety Review Committee. Dose limiting toxicity (DLT) was assessed during the first 28 days. The first 2 dose levels used an accelerated titration design with a cohort size of 1, expanding to 3 if DLT occurred or a Grade 2 adverse event progressed to a higher grade. A maximum of 12 subjects could be treated per dose level.

### Safety assessment

Safety was assessed based on reports of DLTs, Adverse Events (AE), and included clinical laboratory evaluations, vital sign measures, physical examinations, and ECGs. Patients were assessed for AEs at every study visit and as necessary throughout the study. AEs were graded in severity according to the National Cancer Institute CTCAE (version 5.0). Dose-limiting toxicities (DLTs) were defined as death not clearly related to underlying disease or extraneous causes, dose delay > 14 days due to AE, grade ≥ 3 nonhematologic toxicities, grade ≥ 3 thrombocytopenia with clinically significant bleeding, grade ≥ 4 neutropenia or thrombocytopenia lasting > 7 days, or neutropenic fever, any of which were attributed to treatment that occurred in the first 28 days (for exceptions and additional details, see Data Supplement). Patients with DLTs that were ≤ Grade 4 hematological or ≤ Grade 3 nonhematological toxicities could hold treatment until recovery to Grade 1 or baseline, or otherwise resume treatment at a lower dose (unless unresolved within 21 days which would result in permanent discontinuation of treatment). All patients with ≥ Grade 4 nonhematologic toxicities discontinued treatment permanently.

### Clinical activity

Preliminary efficacy measures included post-therapy PSA serum level changes and progression of bony disease as by the Prostate Cancer Working Group 3 (PCWG3) criteria or radiographic response/progression as measurable by RECIST v1.1. Exploratory measures of activity included changes in gene expression of prostate cancer-associated genes (qPCR) and AR protein levels (mean fluorescent intensity) in blood samples isolated from trial subjects.

### CTC capture, qPCR and AR protein quantification

We captured and profiled circulating tumor cells (CTCs) using methods adapted from Schehr et al. [15] (see Supplemental Methods for additional details). This method of CTC capture has been thoroughly developed [16–19], and used across different cancers, including multiple prostate cancer cohorts and trials [1, 19–26]. Briefly, 50 mL of blood was drawn by venipuncture into 10 mL EDTA or CellSave vacutainer tubes. CTCs were then isolated using a custom capture platform as previously described [6, 7, 21, 22]. Gene expression profiling of the captured cells was performed using a multi-plex qPCR platform containing AR (primers for exons AR 1/2 and AR 4/5), AR splice variants (primers for AR V7 and AR V9), AR target genes (primers for TMPRSS2, KLK2, KLK3, FOLH1), and neuroendocrine markers (primers for SYP and CHGA) as previously described [6, 7, 21, 22]. In parallel, AR protein levels were measured from the captured cells using immunofluorescence with an antibody for the AR clone D6F11, with image acquisition and analysis performed at × 20 magnification as previously described [15].

### Statistical analysis

This study was designed to obtain preliminary safety and activity information in the treated populations. Sample sizes in the various dose-escalation and dose-expansion cohorts did not reflect explicit, formal power and type I error considerations. Sample sizes were based on the BOIN design to identify AEs with various assumed underlying prevalence. For safety analysis, all patients who received ONCT-534 were included. Patients were considered for evaluation for response if they had detectable baseline PSA, evidence of measurable disease by RECIST v1.1, or evidence of bony disease by PCWG3. Exploratory endpoints, qPCR and PSA vs qPCR were analyzed via the 2-delta-delta ct method and spearman’s correlation respectively. All statistical tests were two-sided.

## Results

### Patient characteristics and trial conduct

Patient baseline characteristics are listed in Table 1. The majority were White (85.7%) and the remaining were African American or Black (14.3%). A total of 21 patients were enrolled to receive ONCT-534 (Supplementary Fig. 1). Additional patients were intended to be accrued, but due to financial reasons, the trial was stopped early (Supplementary Fig. 2). Patients were assigned to receive ONCT-534 daily at the following doses: 40 mg (*n* = 1), 80 mg (*n* = 1), 160 mg (*n* = 7), 300 mg (*n* = 6), 600 mg (*n* = 3), or 1200 mg (*n* = 3). Median follow-up was 78 days, with an IQR from 60 to 107 days (Supplementary Fig. 2). Following treatment initiation 9 patients withdrew due to disease progression, 1 due to an adverse event, 1 due to withdrawal of consent, 1 due to disease-related death, and the remaining 9 due to study termination by the sponsor. The median baseline pre-ONCT-534 PSA level was 34.1 (range, 1.3–1462.0) ng/mL.

**Table 1:** Summary of patient demographics

Characteristic	All patients (*N* = 21)
**Median age, years (range)**	71 (53–83)
**Male, No. (%)**	21 (100)
**Race, No. (%)**	
American Indian or Alaska Native	0 (0)
Asian	0 (0)
Black or African American	3 (14.3)
Native Hawaiian or Other Pacific Islander	0 (0)
White	18 (85.7)
Other or preferred not to say	0 (0)
Not reported	0 (0)
**EGOG at screening**	
0	7 (33.3)
1	14 (66.6)
**Prostate cancer stage at screening**	
IIIB	1 (4.8)
IIIC	1 (4.8)
IV	6 (28.6)
IVA	2 (9.5)
IVB	6 (28.6)
Unknown	5 (23.8)
**Prior treatment**	
Pharmaseutical therapy	*n* (%)
ARSI	21 (100.0)
Chemo	12 (57.1)
Experimental	6 (28.6)
PSMA-lu	5 (23.8)
PARPi	3 (14.3)
Sip-T	3 (14.3)
Immunotherapy	2 (9.5)
Radium-223	2 (9.5)

### Safety

Of the 21 enrolled subjects there were a total of 108 adverse events (AE) reported throughout the duration of the trial, including 15 serious adverse events (Supplementary Table 1). The most common were anemia (*n* = 6), back pain (*n* = 6), and fatigue (*n* = 6). Of the AEs reported, the investigator considered 24% (*n* = 26) to be related to the study drug, which occurred in 12 of the 21 subjects. There were no DLTs during the first 28 days of treatment. Outside of the window for detecting DLTs, there were 28 Grade 3 or 4 AE across 9 subjects, the most common of which was anemia (*n* = 5). Subject 01005–005 accounted for 28.6% (*n* = 8) of Grade 3 or 4 AE. Only 1 subject, 09001–021, was removed from the trial due to an adverse event (Grade 3 atypical hemolytic uremic syndrome). AE that occurred in > 10% of subjects, and all Grade 3 or 4 AE occurrences are listed in Table 2.

**Table 2:** Treatment-related adverse events observed in > 10% of subjects or > Grade 3

Adverse event	Total (%)	Grade 3 or 4 (%)
Anaemia	6 (29)	5 (24)
Back pain	6 (29)	4 (19)
Fatigue	6 (29)	0 (0)
Acute kidney injury	4 (19)	2 (10)
Nausea	4 (19)	0 (0)
Oedema peripheral	4 (19)	0 (0)
Hyponatraemia	3 (14)	0 (0)
Arthralgia	2 (10)	0 (0)
Blood creatinine increased	2 (10)	0 (0)
Blood lactate dehydrogenase increased	2 (10)	0 (0)
Decreased appetite	2 (10)	0 (0)
Flank pain	2 (10)	0 (0)
Hot flush	2 (10)	0 (0)
Muscle spasms	2 (10)	0 (0)
Muscular weakness	2 (10)	0 (0)
Non-cardiac chest pain	2 (10)	0 (0)
Pain in extremity	2 (10)	0 (0)
Spinal cord compression	2 (10)	2 (10)
**All other Grade 3 or 4 adverse events**		
Atrial fibrillation	1 (5)	1 (5)
Atypical haemolytic uraemic syndrome	1 (5)	1 (5)
Blood alkaline phosphatase increased	1 (5)	1 (5)
Extradural neoplasm	1 (5)	1 (5)
Haematuria	1 (5)	1 (5)
Hyperglycaemia	1 (5)	1 (5)
Hyperinsulinaemia	1 (5)	1 (5)
Hypocalcaemia	1 (5)	1 (5)
Hypotension	1 (5)	1 (5)
Lactic acidosis	1 (5)	1 (5)
Lumbosacral plexopathy	1 (5)	1 (5)
Paraesthesia	1 (5)	1 (5)
Retinal artery occlusion	1 (5)	1 (5)
Urinary tract obstruction	1 (5)	1 (5)
Urosepsis	1 (5)	1 (5)

### Clinical response

**Fig. 1:**
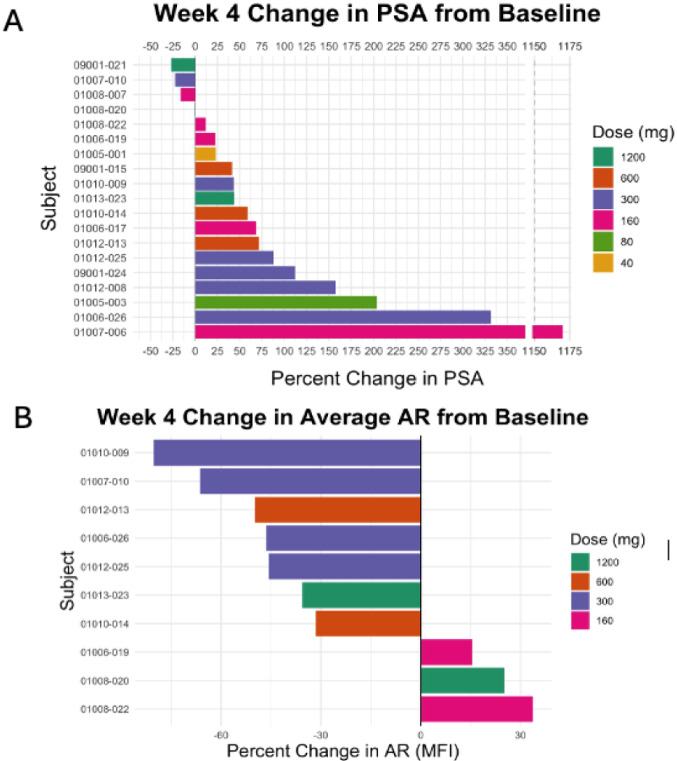
Changes in protein biomarkers following treatment with ONCT-534 between screening and week 4. **A** Percent change in early PSA kinetics, from baseline to week 4. **B** Percent change in AR protein from screening to week 4. Measured via mean fluorescent mean intensity (MFI) from blood samples stained with anti-AR antibody

Results from imaging studies were available on seven patients (Supplementary Fig. 3). In evaluating response based on RECIST v1.1 and Prostate Cancer Working Group 3 (PCWG3) criteria, one patient was not evaluable, five had progressive disease, and one patient with bone-only metastases had a best response of stable disease. Of 19 evaluable patients, three demonstrated decreased PSA measures at week 4 compared to baselines (Fig. 1A) but no subjects obtained a > 50% decrease in PSA from baseline (PSA_50_). One patient achieved a PSA_50_ around the time of study closure after the data lock period. It should be noted that several patients were preliminarily terminated and thus did not have sufficient follow-up time to fully assess for response.

### Biomarker response: AR protein and qPCR

We next wanted to directly assess the effect of ONCT-534 on AR protein levels in an exploratory analysis of the liquid biopsy data (Supplementary Fig. 3). Ten subjects had baseline AR protein data, and showed a median change in AR protein of − 40.59% at week 4 (interquartile range, − 48.96% to 3.77%). Seven patients demonstrated a decrease in AR Protein > 30% at week 4 (Fig. 1B). All patients receiving the 300 mg or 600 mg dose of ONCT-534 (who had AR protein data) demonstrated a decrease in AR protein at week 4. No patient who received the 160 mg dose of ONCT-534 had a decrease in AR protein.

**Fig. 2:**
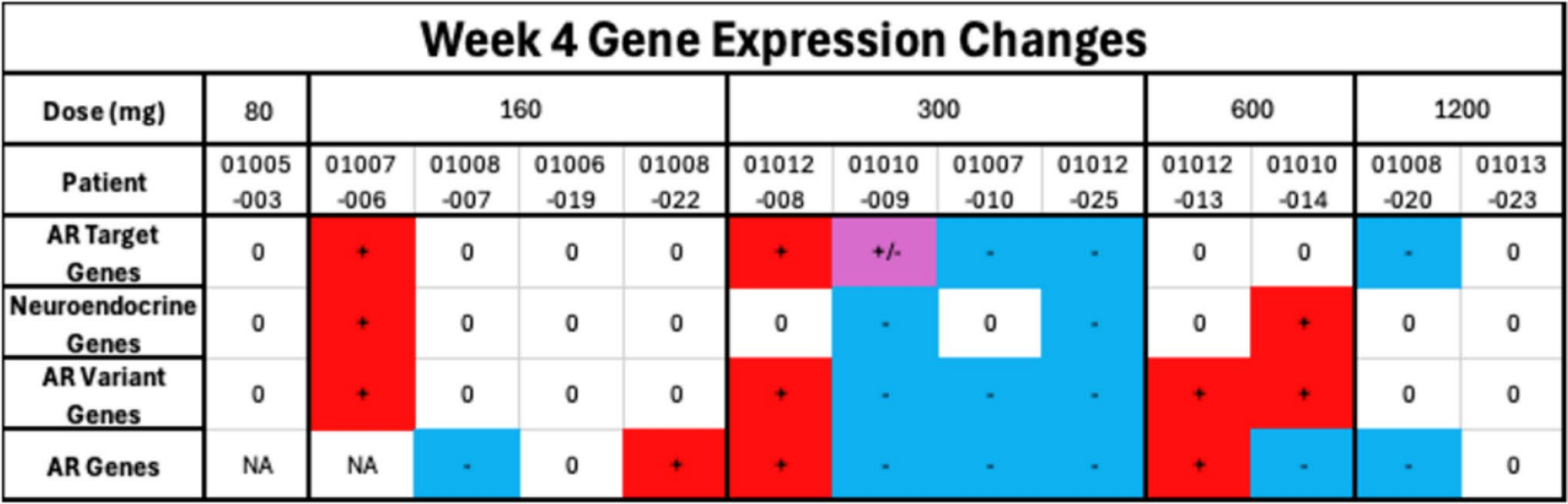
Gene expression changes at week 4 relative to screening, following treatment with ONCT-534. 13 patients had screening and week 4 gene expression data. Gene expression measured via qPCR from circulating tumor cells (CTCs). AR Target Genes include KLK2, KLK3, FOLH1, and TMPRSS2. Neuroendocrine Genes include SYP (CHGA was measured but never detected in any samples), AR Variant Genes include AR V7 and AR V9, and AR Genes include AR 1/2 and AR 4/5. + (in red) = increased expression of at least 1 gene in that category.—(in blue) = decreased expression of at least 1 gene in that category. ± (in purple) = increased and decreased expression of genes in that category. 0 = no detectable change in gene expression in that gene category. NA = no data available for that gene category

**Fig. 3:**
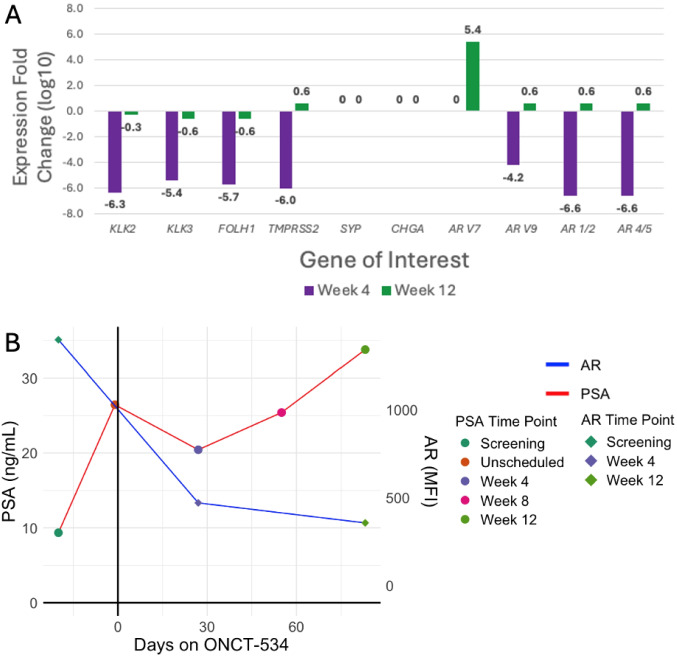
Example subject, 01007–010 (300 mg), with biomarker changes following ONCT-534 treatment. **A** Individual gene expression changes, measured via qPCR from CTC, at week 4 and week 12 as compared to screening. **B** AR protein levels (blue line) and PSA levels (red line) before and following treatment with ONCT-534

Thirteen subjects had qPCR data, at baseline and week 4, measuring AR target genes (KLK2, KLK3, FOLH1, and TMPRSS2), neuroendocrine genes (SYP and CHGA), AR variants (AR V7 and ARV9), and AR genes (AR 1/2 and AR 4/5). Within the 300 mg ONCT-534 dose group, 3 subjects demonstrated decreased expression of AR target genes, AR variant genes, and AR genes (Fig. 2), and these 3 subjects (01010–009, 01007–010, and 01012–025) were also 3 of the subjects with decreased AR protein levels (Fig. 1B). Specifically, subject 01007–010 demonstrated decreased PSA, AR protein levels, AR target genes, AR variant genes, and AR genes at week 4, relative to baseline (Fig. 3). One patient in the 160 mg dose group and one patient in the 600 mg dose group demonstrated an increased expression of SYP at week 4, a marker associated with the neuroendocrine differentiation (Fig. 2). Other dose groups did not demonstrate any clear patterns of changes in AR target, neuroendocrine, AR variant, or AR genes.

**Fig. 4:**
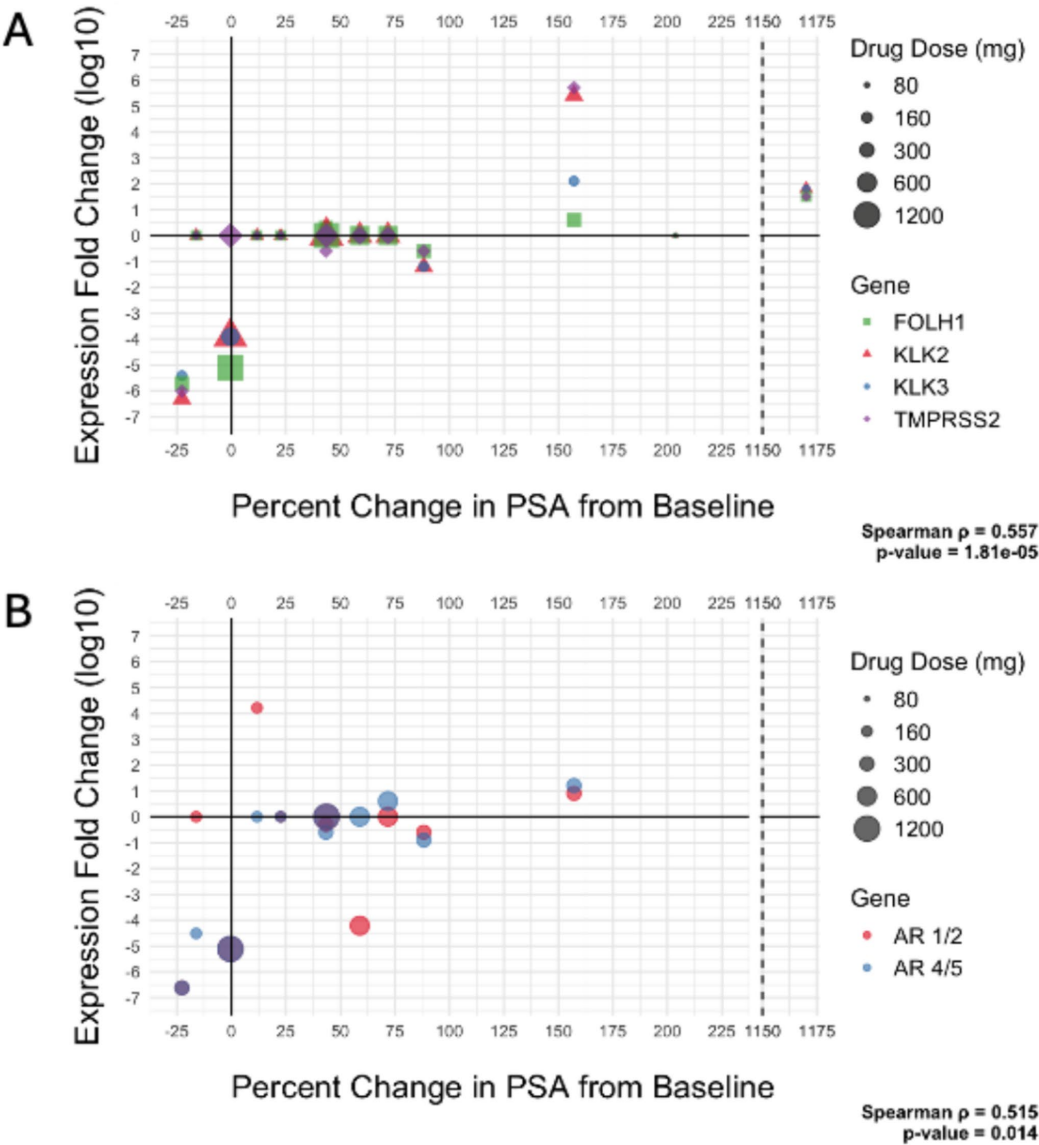
PSA vs gene expression changes with associations at week 4 compared to screening. **A** PSA vs AR Target Gene expression (FOLH1, KLK2, KLK3, and TMPRSS2); Spearman = 0.557 and p-value = 1.81e-05. **B** PSA vs AR Gene expression (AR 1/2 and AR 4/5); Spearman = 0.515 and p-value = 0.014

PSA levels demonstrated positive associations with expression of AR target genes (Spearman rho = 0.557, *p* = 1.81e-05) and AR genes (Spearman rho = 0.515, *p* = 0.014), but no association with expression of AR gene variants or neuroendocrine genes (Fig. 4 and Supplementary Fig. 4 respectively).

## Discussion

The DAARI ONCT-534 had no DLTs in the first 28 days and demonstrated biological effectiveness at reducing AR protein levels in mCRPC patients, providing the first proof of concept in human patients that DAARIs can degrade AR and block AR signaling. Unfortunately, this did not translate into robust clinical responses, and the trial was stopped early due to financial reasons. This potentially impacts the interpretation of the clinical outcomes, as it is possible that there are patients who were on the drug at the trial closure that may have demonstrated a clinical benefit had the trial continued, given preclinical models showed AR protein reduction preceded AR gene inhibition and tumor response [27]. It is additionally possible that given there were 28 grade 3 or 4 AEs beyond the DLT window, including 6 anemic events, there could be accumulative toxicity that was censored by premature trial closure.

While this trial was not powered to assess efficacy, the lack of PSA or radiographic response may be due to a sufficiently high residual pool of AR allowing the tumor cells to continue to grow and proliferate. Some patients showed discordant signals (decreased AR protein but increased AR target genes/variants), which could suggest in these patients, the undegraded AR pool is sufficient for cancer progression or the tumor is driven by AR-independent mechanisms. For example, two patients demonstrated SYP expression, often associated with neuroendocrine differentiation, which could explain their resistance to AR degradation therapy.

Other ongoing AR degrader trials have shown more promise, particularly when targeting patients with certain ligand binding domain (LBD) mutations. LBD mutations often result in gain-of-function, enabling AR to be activated by former antagonists (the F877L mutation converts enzalutamide from AR inhibitor into an agonist) [28] or even in response to non-androgenic steroid hormones [29, 30]. Initial results from a phase 1/2 study of ARV-766 demonstrated a PSA_50_ in 50% of PSA-evaluable mCRPC patients (*n* = 28) with LBD mutations [31]. Another ongoing trial, a phase 1/2 study of ARV-110, showed in a preliminary analysis of patients with at least 1 month of PSA follow-up, patients with the LBD mutation AR T878X/H8757Y achieved a PSA_50_ in 46% of patients (*n* = 28), while patients with wild type LBD achieved a PSA_50_ in only 11% of patients (*n* = 44), and patients with the LBD mutation L702H/AR-V7 achieved a PSA_50_ in only 4% of patients (*n* = 25) [32]. Given the short duration of AR protein level decreases and lack of clinical response, even at higher doses, a DAARI with more potent NTD inhibition than ONCT-534 and/or tumor biomarker-stratification may be needed to achieve meaningful clinical outcomes. While pharmokinetics were not evaluated in this study, poor bioavailability could also serve as an explanation for the limited responses observed in this study.

The preliminary response rates in these other trials suggest that AR degraders still have potential as a therapeutic strategy in selected populations. Our study also highlights the importance of biomarkers in the assessment of AR degrader effects in human trials. CTC assessment of serial AR protein and target gene expression may help elucidate which patients are poised to benefit from AR degraders, as well as why some patients do not. Measuring AR protein levels across other trials may help determine whether, and if so, what degree of AR protein degradation is necessary from an AR degrader to achieve a consistent clinical response.

## Supplementary Information

Below is the link to the electronic supplementary material.

Supplementary file1 (DOCX 822 kb)

Supplementary file2 (DOCX 17 kb)

## Data Availability

No datasets were generated or analysed during the current study.
