# Supplementary material for: Phase I multi-center clinical and biomarker study of the dual-action androgen receptor inhibitor ONCT-534
Source: Invest New Drugs. 2026 Mar 3;44(2):103–12. doi: 10.1007/s10637-026-01600-8 (PMC13292475; doi:10.1007/s10637-026-01600-8)
Supplement: Supplementary file 1 — Supplementary file1 (DOCX 822 kb) [file 10637_2026_1600_MOESM1_ESM.docx]

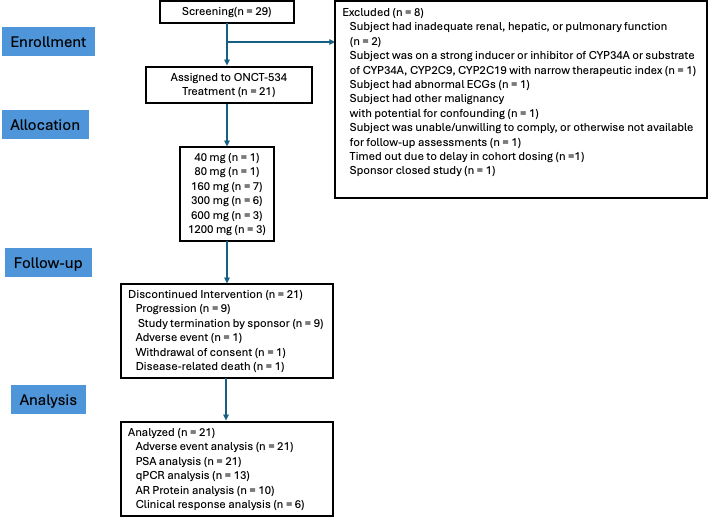


**Supplementary Figure 1.** Consort diagram of ONCT-534 trial.


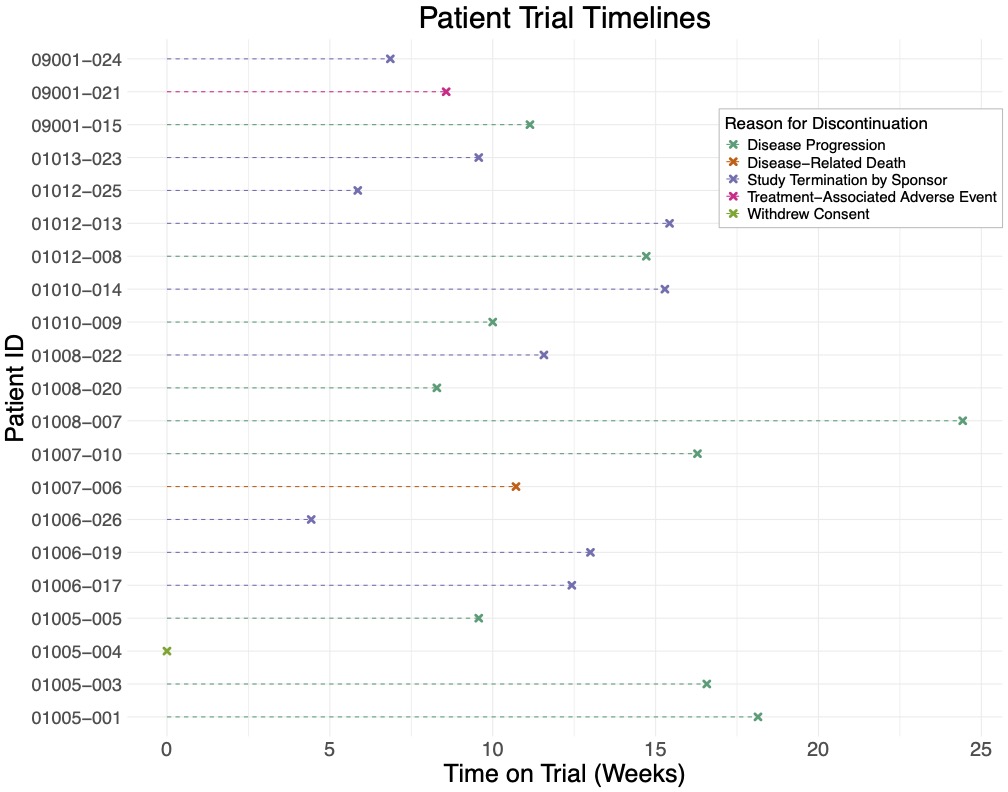


**Supplementary Figure 2.** Time on trial for individual patients. The X’s to indicate individual patient trial stoppage time and the reason for trial discontinuation.


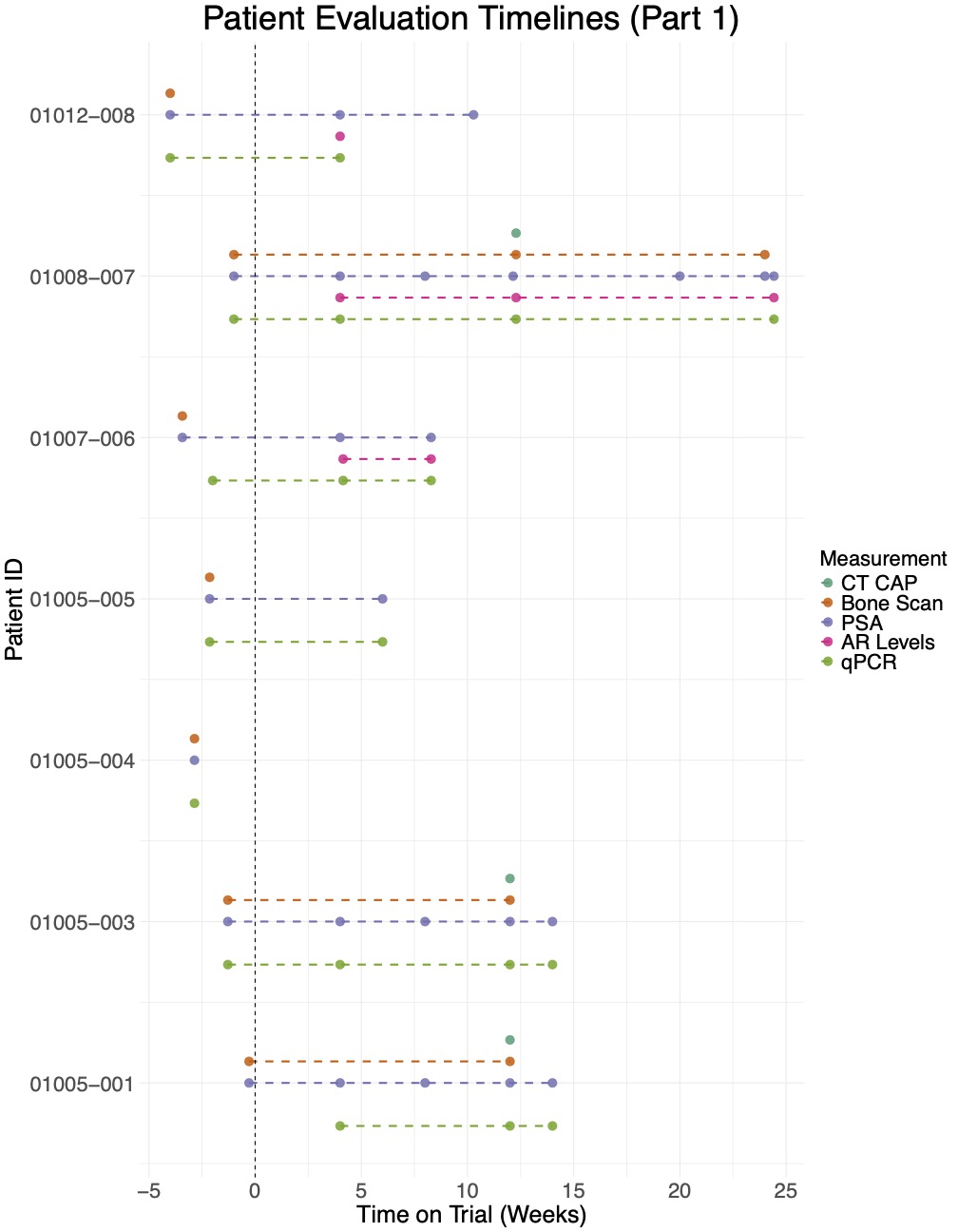


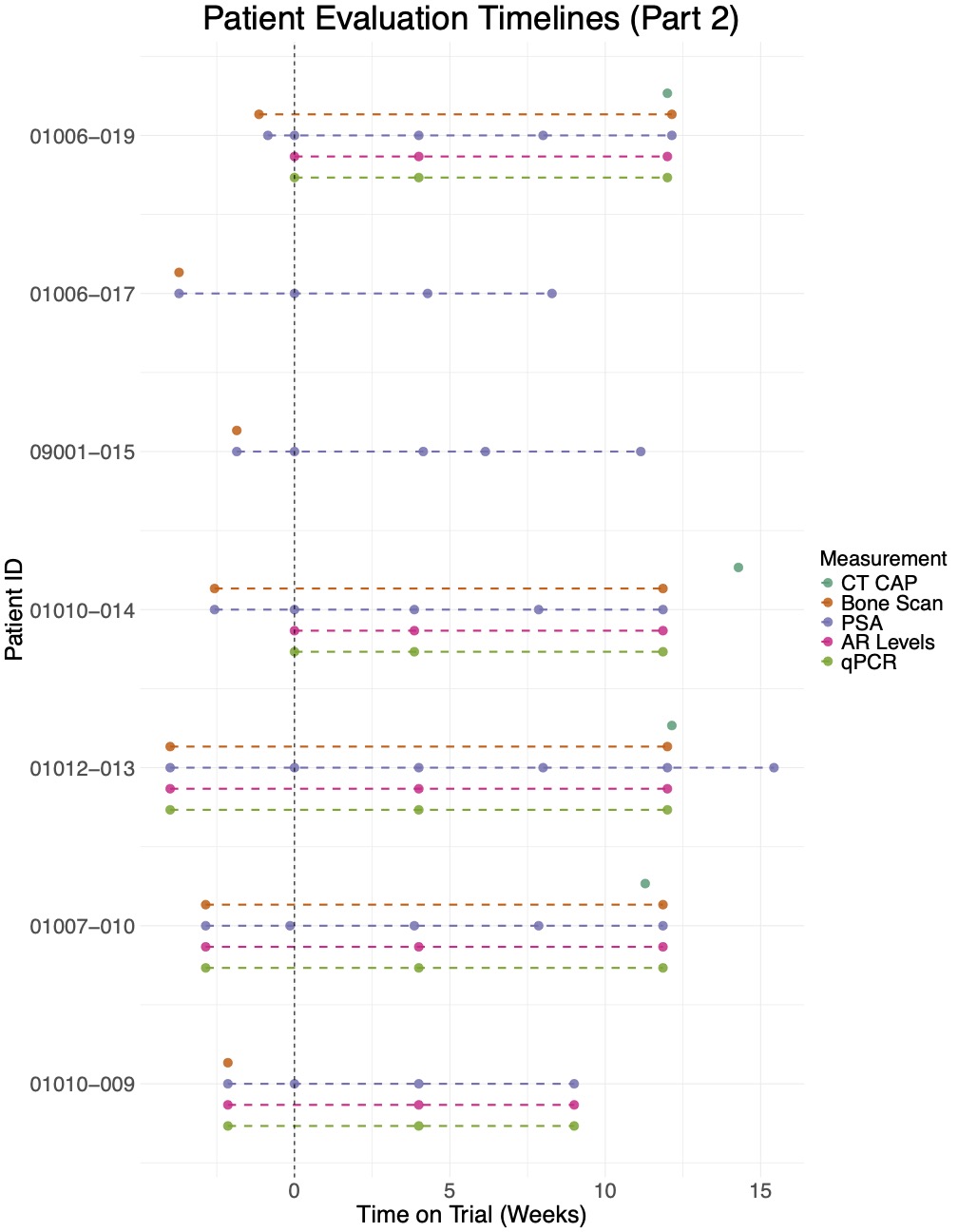


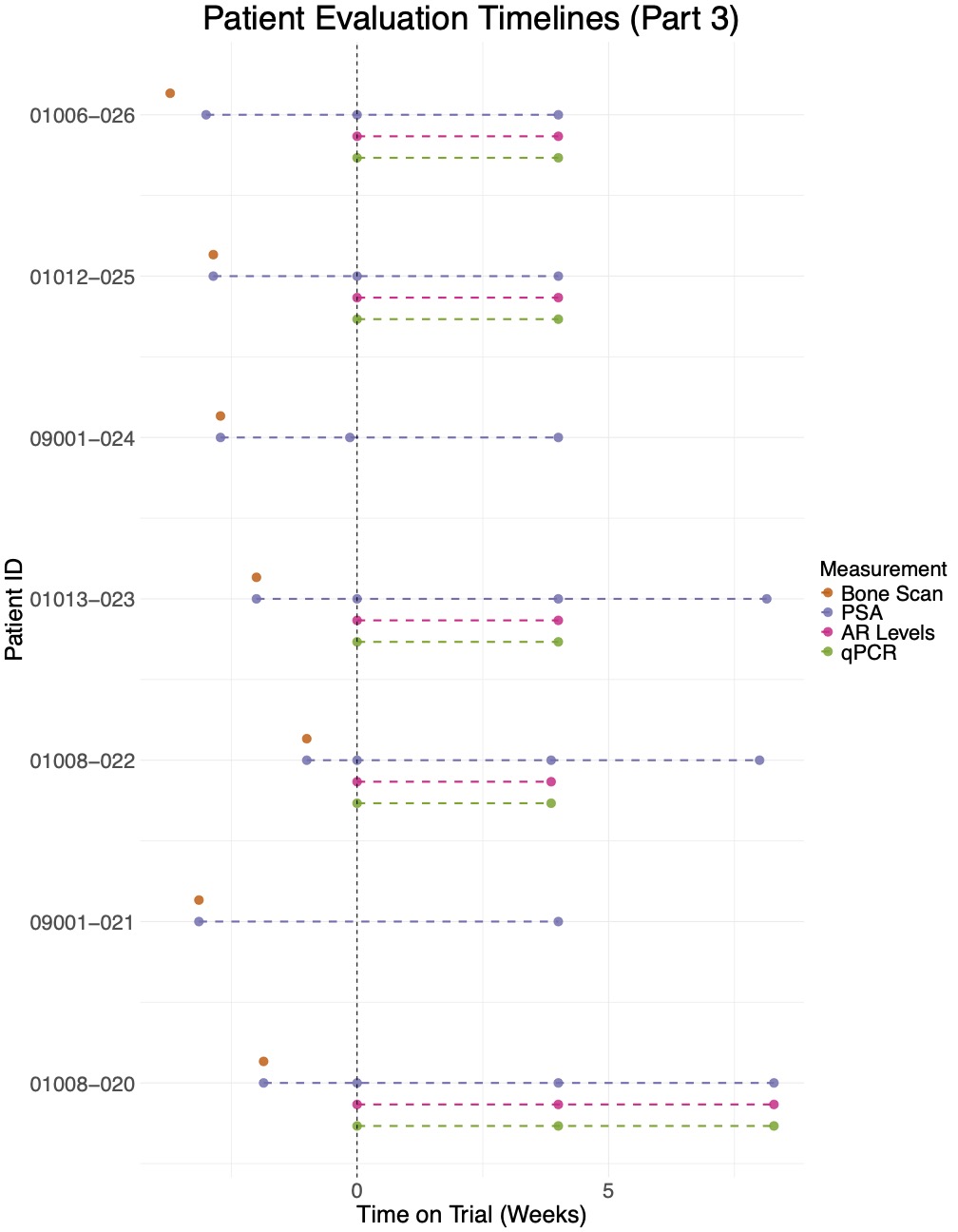


**Supplementary Figure 3.** Patient evaluation timelines showing timepoints at which different samples were collected from each patient, including CT chest-abdomen-pelvis (CT CAP), bone scans, blood for PSA, blood for AR levels, and blood for qPCR. Due to a combination of missed visits and early closure of the trial, multiple patients are missing data at different time points.


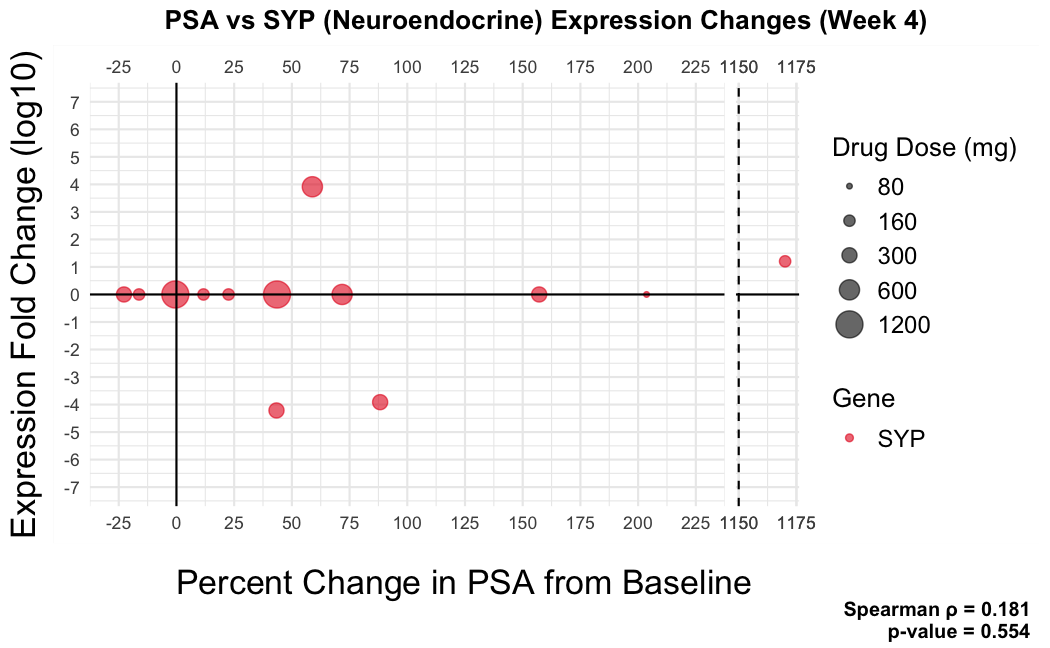

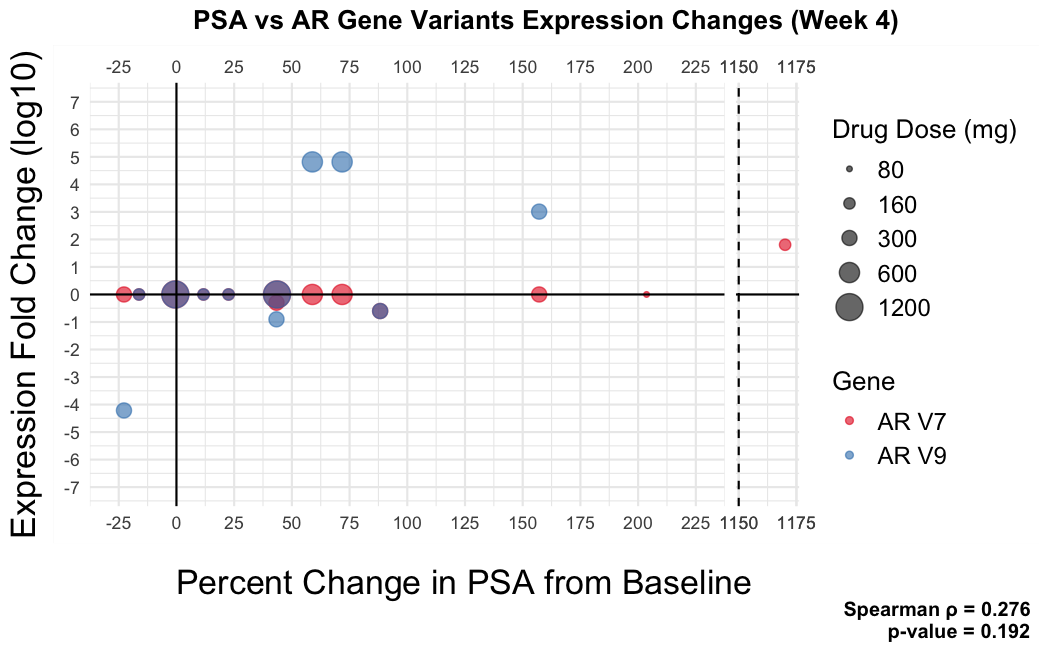


**Supplementary Figure 4.** PSA vs gene expression changes without significant correlations at week 4 compared to screening. (A) PSA vs AR Variant Gene expression (AR V7 and AR V9); Spearman = 0.276 and p-value = 0.192. (B) PSA vs Neuroendocrine Gene expression (SYP); Spearman = 0.276 and p-value = 0.554.

B

A

| **Serious Adverse Events** | |
| --- | --- |
| **Adverse Event** | **n (%)** |
| Acute kidney injury | 3 (14) |
| Back pain | 3 (14) |
| Anaemia | 1 (5) |
| Atypical haemolytic uraemic syndrome | 1 (5) |
| Lactic acidosis | 1 (5) |
| Non-cardiac chest pain | 1 (5) |
| Seroma | 1 (5) |
| Spinal cord compression | 1 (5) |
| Thromboembolic event | 1 (5) |
| Urinary tract obstruction | 1 (5) |
| Urosepsis | 1 (5) |

**Supplementary Table 1.** All serious adverse events including any deaths, life-threatening medical events, medical events requiring hospitalization or extending an existing hospitalization, or otherwise resulting in substantial disruption to a patient’s normal life function.

| **List of prior cancer drugs received by patients before starting on ONCT-534** | |
| --- | --- |
| **Subject** | **Drug Name** |
| 01005-001 | 1 - LEUPRORELIN - 09/Dec/2022 |
| 01005-001 | 2 - NUBEQA - 30/Jun/2023 |
| 01005-003 | 1 - LEUPRORELIN - 22/May/2012 |
| 01005-003 | 2 - LEUPRORELIN - 16/Sep/2014 |
| 01005-003 | 3 - LEUPRORELIN - 14/Mar/2017 |
| 01005-003 | 4 - LEUPRORELIN - 30/Jan/2020 |
| 01005-003 | 5 - LEUPRORELIN - 09/Sep/2021 |
| 01005-003 | 6 - NUBEQA - 26/May/2022 |
| 01005-004 | 1 - ELIGARD - 30/Apr/2015 |
| 01005-004 | 2 - BICALUTAMIDE - 27/Mar/2017 |
| 01005-004 | 3 - ELIGARD - 11/Mar/2019 |
| 01005-004 | 4 - ENZALUTAMIDE - UN/Jun/2020 |
| 01005-004 | 5 - ELIGARD - UN/Mar/2022 |
| 01005-004 | 6 - ORGOVYX - 23/Nov/2023 |
| 01005-005 | 1 - ELIGARD - 01/Nov/2022 |
| 01005-005 | 2 - ABIRATERONE - UN/Nov/2022 |
| 01007-006 | 1 - Doxorubicin (Adriamycin) - 03/Mar/2014 |
| 01007-006 | 2 - Docetaxel (taxotere) - 03/Mar/2014 |
| 01007-006 | 3 - Estramustine - 03/Mar/2014 |
| 01007-006 | 4 - Degarelix (Firmagon) - 03/Mar/2014 |
| 01007-006 | 5 - Leuprorelin (Lupron) - 21/Jul/2014 |
| 01007-006 | 6 - Enzalutamide (Xtandi) - 21/Jun/2019 |
| 01007-006 | 7 - Abirateron acetate (Zytiga) - 16/Jul/2020 |
| 01007-006 | 8 - Apalutamide - 01/Oct/2020 |
| 01007-006 | 9 - Docetaxel (taxotere) - 06/Jan/2021 |
| 01007-006 | 10 - Olaparib (Lynparza) - 22/Sep/2021 |
| 01007-006 | 11 - Cabazitaxel - 27/Jun/2022 |
| 01007-006 | 12 - NGM831 - 31/Aug/2022 |
| 01007-006 | 13 - Pluvicto (Lutetium (177Lu) vipivotide tetraxetan) - 07/Oct/2022 |
| 01007-006 | 14 - Darolutamide - 23/Aug/2023 |
| 01007-006 | 15 - Docetaxel - 23/Aug/2023 |
| 01008-007 | 1 - docetaxel - 18/Oct/2022 |
| 01008-007 | 2 - abiraterone - UN/May/2022 |
| 01012-008 | 1 - Zytiga - un/May/2019 |
| 01012-008 | 2 - Lupron - un/Jun/2019 |
| 01012-008 | 3 - Provenge - un/Jul/2021 |
| 01012-008 | 4 - Docetaxel - 11/Jul/2022 |
| 01012-008 | 5 - Cabazitaxel - 05/Dec/2022 |
| 01012-008 | 7 - Leuprolide - 28/Mar/2023 |
| 01012-008 | 6 - Enzalutamide - 14/Sep/2023 |
| 01010-009 | 1 - Bicalutamide - 23/Mar/2020 |
| 01010-009 | 2 - Leuprolide - 23/Mar/2020 |
| 01010-009 | 3 - Docetaxel - 13/Jul/2020 |
| 01010-009 | 4 - Abiraterone - 17/May/2021 |
| 01010-009 | 5 - Enzalutamide - 3/Aug/2021 |
| 01010-009 | 6 - Olaparib - 2/Jun/2022 |
| 01010-009 | 7 - Xofigo - 2/Jun/2022 |
| 01010-009 | 8 - Pluvicto - 17/Oct/2023 |
| 01007-010 | 1 - Leuprolide - 28/Apr/2017 |
| 01007-010 | 2 - Bicalutamide - 25/Aug/2017 |
| 01007-010 | 3 - Darolutamide (Nubeqa) - un/UNK/2020 |
| 01007-010 | 4 - Sipuleucel-T (Provenge) - 30/Sep/2022 |
| 01007-010 | 5 - Trelstar - 26/Jul/2022 |
| 01007-010 | 6 - Docetaxel - 10/Apr/2023 |
| 01007-010 | 7 - Leuprolide - 12/Jun/2023 |
| 01012-013 | 1 - Eligard (Leuprolide acetate) - 11/Jul/2022 |
| 01012-013 | 2 - Bicalutamide (Casodex) - un/Jun/2022 |
| 01012-013 | 4 - Apalutamide (Erleada) - 02/Aug/2022 |
| 01010-014 | 1 - Lupron - 18/Jul/2019 |
| 01010-014 | 2 - Leuprolide - 16/Sep/2022 |
| 01010-014 | 3 - Enzalutamide - 13/Feb/2023 |
| 09001-015 | 1 - Triptorelin - UN/May/2017 |
| 09001-015 | 2 - Bicalutamide - UN/Jun/2017 |
| 09001-015 | 8 - Docetaxel - UN/Jul/2017 |
| 09001-015 | 3 - Triptorelin - UN/Oct/2020 |
| 09001-015 | 4 - Abiraterone & Prednisone - UN/Apr/2021 |
| 09001-015 | 5 - Abiraterone & Dexamethasone - UN/Jan/2022 |
| 09001-015 | 6 - Cabazitaxel - UN/Nov/2022 |
| 09001-015 | 7 - Enzalutamide - 06/Nov/2023 |
| 01006-017 | 1 - ELIGARD - 27/Dec/2019 |
| 01006-017 | 2 - RELUGOLIX - 04/Jan/2022 |
| 01006-017 | 3 - DAROLUTAMIDE - 12/May/2022 |
| 01006-019 | 1 - LUPRON - 22/Sep/2008 |
| 01006-019 | 2 - BICALUTAMIDE - 22/Sep/2008 |
| 01006-019 | 3 - SIPULEUCEL-T - 17/May/2012 |
| 01006-019 | 4 - GTX-758 - 13/Mar/2013 |
| 01006-019 | 5 - ABIRATERONE - 21/Jul/2013 |
| 01006-019 | 7 - PEMBROLIZUMAB - 02/May/2017 |
| 01006-019 | 8 - ENZALUTAMIDE - 02/May/2017 |
| 01006-019 | 9 - DOCETAXEL - 26/Dec/2017 |
| 01006-019 | 10 - CABAZITAXEL - 05/Jan/2021 |
| 01006-019 | 11 - 177LU-PSMA617 - 22/Jun/2022 |
| 01008-020 | 1 - Casodex - UN/Dec/2018 |
| 01008-020 | 2 - Lupron - UN/Dec/2018 |
| 01008-020 | 3 - Prostvac - UN/Jan/2019 |
| 01008-020 | 4 - Docetaxel - UN/Jan/2019 |
| 01008-020 | 5 - Investigational Drug NHS-IL12 - UN/Aug/2019 |
| 01008-020 | 6 - Zytga - 31/Aug/2020 |
| 01008-020 | 7 - Xtandi - 28/Mar/2022 |
| 01008-020 | 8 - Cabazitaxel - 24/Jan/2023 |
| 01008-020 | 9 - Investigational Drug - NPX887 - 6/May/2024 |
| 09001-021 | 1 - Celecoxib - 14/Oct/2009 |
| 09001-021 | 2 - Bicalutamide - 11/Mar/2015 |
| 09001-021 | 3 - Ipatasertib/Placebo - 24/Apr/2018 |
| 09001-021 | 4 - Abiraterone - 24/Apr/2018 |
| 09001-021 | 5 - Docetaxel - 03/Dec/2021 |
| 09001-021 | 6 - Cabazitaxel - 12/Dec/2022 |
| 09001-021 | 7 - PSMA-CD3 - UN/Sep/2023 |
| 09001-021 | 8 - EVICTION-2 - 26/Feb/2024 |
| 01008-022 | 1 - Leuprolide - 25/Aug/2021 |
| 01008-022 | 2 - Abiraterone - 17/Jun/2022 |
| 01013-023 | 1 - zoladex - UN/Jan/2011 |
| 01013-023 | 2 - lupron - UN/UNK/2012 |
| 01013-023 | 3 - darolutamide - UN/Feb/2022 |
| 09001-024 | 1 - Leuprorelin - UN/UNK/2016 |
| 09001-024 | 2 - Docetaxel - UN/Apr/2016 |
| 09001-024 | 3 - Abiraterone & Prednisone - UN/May/2018 |
| 09001-024 | 4 - Cellcentric - UN/Jan/2019 |
| 09001-024 | 5 - Cabazitaxel - UN/Dec/2019 |
| 09001-024 | 6 - Radium-223 - UN/Sep/2021 |
| 09001-024 | 7 - Crescendo Phase I Trial - UN/Mar/2022 |
| 09001-024 | 8 - Lutetium - UN/Jan/2023 |
| 01012-025 | 1 - Bicalutamide - un/UNK/2014 |
| 01012-025 | 2 - Abiraterone - un/UNK/2015 |
| 01012-025 | 3 - Docetaxel - 02/May/2016 |
| 01012-025 | 4 - Enzalutamide - 03/Sep/2018 |
| 01012-025 | 5 - Cabazitaxel - 04/Aug/2020 |
| 01012-025 | 6 - Olaparib - 20/Mar/2022 |
| 01012-025 | 3 - Docetaxel - 02/May/2016 |
| 01012-025 | 8 - M1774 - 06/Jul/2023 |
| 01012-025 | 9 - Avelumab - 06/Jul/2023 |
| 01012-025 | 10 - BMS-986460 - 10/Jan/2024 |
| 01012-025 | 11 - ABBV-969 - 04/Apr/2024 |
| 01006-026 | 1 - LUPRON - 11/Apr/2017 |
| 01006-026 | 2 - ENZALUTAMIDE - 19/Jul/2019 |
| 01006-026 | 3 - ABIRATERONE - 13/Sep/2019 |
| 01006-026 | 5 - DOCETAXEL - 30/Aug/2021 |
| 01006-026 | 6 - PLUVICTO - 30/Aug/2023 |
| 01006-026 | 7 - APALUTAMIDE - 11/Oct/2023 |

**Supplementary Table 2. List of prior cancer drugs received by each patient prior to starting treatment with ONCT-534**
