# Supplementary material for: Phase I multi-center clinical and biomarker study of the dual-action androgen receptor inhibitor ONCT-534
Source: Invest New Drugs. 2026 Mar 3;44(2):103–12. doi: 10.1007/s10637-026-01600-8 (PMC13292475; doi:10.1007/s10637-026-01600-8)
Supplement: Supplementary file 2 — Supplementary file2 (DOCX 17 kb) [file 10637_2026_1600_MOESM2_ESM.docx]

**Patient Sample Collection**

To harvest PBMCs, 50 mL of blood was drawn by venipuncture into 10 mL EDTA or CellSave vacutainer tubes, each 15 mL volume of blood was diluted 1:1 with PBS (Life Technologies), underlaid with 10 mL of ficoll-paque (General Electric, Boston, MA), and spun for 20 min at 1000 g in a swing-bucket rotor centrifuge. PBMCs were washed twice with ambient PBS to remove platelets prior to subsequent manipulation. For mRNA biomarkers, PBMCs isolated from EDTA tubes were further depleted of CD45+ and CD235a+ cells using a standard LS MACS column (Miltenyi, Bergisch Gladbach, Germany) per manufacturer’s guidelines, with the exception of limiting the volume of CD235a beads per LS column to no more than 25uL. For protein biomarkers, PBMCs isolated from CellSave tubes were fixed for 20min at 5C with cytofix (BD), then washed with 50mL PBS.

**ESP Cell Capture**

To facilitate magnetic capture and manipulation of target cells, Streptavidin-blocked SeraMag SpeedBead Paramagnetic Particles (PMPs) (General Electric) were conjugated to biotinylated EpCAM antibody (BAF960, R&D Systems, Minneapolis, MN) by first washing the PMPs twice with 0.1% Tween in PBS then adding 2 uL of antibody. After incubating 20 min at room temperature with constant vortex agitation, beads were washed twice with 10% FBS in PBS, then allowed to bind to CD45-depleted or fixed patient sample PBMCs for 20 min under constant rotation at 4 C in a total volume of 500 uL of 10% FBS. For automated sample processing, cells bound to PMPs were transferred to the input wells of the ExtractMan extraction plates (100 uL final volume version) (Gilson, Middleton, WI). Cells were pulled from the input well into one large wash well (250 uL volume of 10% FBS) using the opposing magnetic forces in the hovering magnetic pipette head attachment, and the underlying magnetic bar floating within the plate platform, then mixed with slow pipette mixing to maximize viability^5^.

**mRNA Extraction and qRT-PCR**

For mRNA biomarkers, mRNA extraction plates and strips (Gilson, Inc) were first briefly submerged in RNAse away (Life Tech), rinsed with nuclease-free distilled water (Thermofisher Scientific), and allowed to air dry. mRNA was extracted from captured CTCs by incubating them for 5 minutes at room temperature (RT) in 10% Lysis Binding Buffer (LBB) (Dynabeads mRNA DIRECT, Thermofisher Scientific) diluted in PBS containing 10uL Dynabeads Oligo(dT)25 beads (washed once with 10% LBB prior to use). The PMPs were collected then transferred through sequential washes of wash buffer A (WBA), 1% PBST and PBS prior to being eluted into molecular grade nuclease-free water (Promega, Inc). mRNA was reverse transcribed to cDNA with High-Capacity RNA-to-cDNA kit (Applied Biosystems), PreAmplified for 14 cycles with TaqMan PreAmp Master Mix (Applied Biosystems), and diluted 1:20 in TE buffer (Promega) prior to qPCR using TaqMan Assays (ThermoFisher).

**Androgen Receptor Staining and Measurement**

Cells were pulled into a subsequent reagent well containing 1:70 concentrations in 10% FBS of extracellular fluorescent antibodies against EpCAM (vendor), CD14 (vendor), CD45 clone HI30 (Biolegend), CD34 clone 581 (Biolegend) and CD66b clone G10F5 (Biolegend), along with Hoechst 33342 pre-diluted 1:25 in 10% FBS (Life Technologies), and allowed to incubate at RT for 20 min. Cells were then pulled into Foxp3 perm/wash (eBioscience), and incubated for 15 min at RT. Cells were then pulled into foxp3 perm/wash containing 1:70 concentration of the intracellular fluorescent antibody against pan cytokeratin (pCK) clone C-11 (Santa Cruz Biotechnology) and 1:140 concentration of unconjugated AR clone D6F11 (Cell Signaling Technology) and incubated for 20 min at RT. Cells were then pulled into a 10% FBS wash well, then into the secondary stain containing fluorescent donkey anti-rabbit clone Poly4064 (Biolegend) at 1:140 in foxp3 perm/wash for 20min at RT. Cells were pulled through 2 additional 10%FBS wash wells and then into a final well of PBS prior to imaging. Image acquisition and analysis was performed at 20x magnification as previously described^24^.
